# Profound Hypotension before Aortic Clamping Can Exacerbate Spinal Cord Ischemic Injury after Aortic Surgery in Rats

**DOI:** 10.3390/jcm9113395

**Published:** 2020-10-23

**Authors:** Chang-Hoon Koo, Jung-Hee Ryu, Jin-Young Hwang, Jin-Hee Kim, Hyun-Jung Shin, Sung-Hee Han

**Affiliations:** 1 Department of Anesthesiology & Pain medicine, Seoul National University Bundang Hospital, Seongnam 13620, Korea; vollock9@gmail.com (C.-H.K.); jinaryu74@gmail.com (J.-H.R.); anesing1@snu.ac.kr (J.-H.K.); medidoc@nate.com (H.-J.S.); 2 Department of Anesthesiology & Pain medicine, Seoul National University College of Medicine, Seoul 03080, Korea; mistyblue15@naver.com; 3 Department of Anesthesiology & Pain medicine, SMG-SNU Boramae Medical Center, Seoul 07061, Korea

**Keywords:** aortic surgery, ischemia-reperfusion injury, hypotension, spinal cord injury

## Abstract

Spinal cord ischemia is one of the most serious complications of aortic repair in patients with acute aortic syndrome. However, the effect of hypotension before aortic clamping on spinal cord injury has not been documented. A total of 48 male Sprague-Dawley rats were randomly divided into four groups: the sham group; control group (mean arterial pressure (MAP) < 90% of baseline value before aortic clamping); mild hypotension group (MAP < 80%); and profound hypotension group (MAP < 60%). Spinal cord ischemia was induced using a balloon-tipped catheter placed in the descending thoracic aorta. Neurological function of the hind limbs was evaluated for seven days after reperfusion and recorded using a motor deficit index (MDI). The spinal cord was then harvested for histopathological examination and evaluation of oxidative stress and inflammation. The profound hypotension group demonstrated a significantly higher MDI 48 h post-reperfusion and lower number of normal motor neurons than the other groups (*p* < 0.001). The levels of tissue malondialdehyde and tumor necrosis factor-α (TNF-α) were also significantly increased in the profound hypotension group compared with other groups. Profound hypotension before aortic clamping can aggravate neurologic outcomes after aortic surgery by exacerbating neurologic injury and reducing the number of normal motor neurons.

## 1. Introduction

Spinal cord ischemia is one of the most serious complications of aortic repair in patients with acute aortic syndrome, occurring in approximately 3–16% of cases following open surgery or thoracic endovascular aortic repair (TEVAR) [1,2,3]. Spinal cord ischemia following aortic repair is caused by numerous factors [4]. Systemic hypotension that occurs during or after the aortic clamping is a well-documented risk factor of spinal cord ischemia following aortic surgery [5,6,7,8]. 

Before aortic clamping, controlled hypotension is usually performed to prevent aortic rupture and to facilitate the surgical procedure in patients with aortic syndrome [9,10]. However, up to 47% of controlled hypotension could be associated with unintended hemodynamic instability [11] and the effect of degree of hypotension during the pre-clamping period on spinal cord ischemic injury has not been documented in acute aortic syndrome. In cerebral ischemic injury, severe hypotension before reperfusion aggravates neurological injury [12,13]. Thus, it could be hypothesized that excessive hypotension before aortic clamping may also aggravate neurological injury following spinal cord ischemia-reperfusion. To identify the effect of degrees of hypotension before aortic clamping, we performed an animal study using a rat model of spinal cord ischemia-reperfusion injury. Various degrees of hypotension were deliberately induced before aortic clamping, and the extent of spinal ischemia-reperfusion injury was compared, including postoperative neurological deficit. 

## 2. Materials and Methods

### 2.1. Animal Care

The protocol of the current study was approved by the Institutional Animal Care and Use Committee of Seoul National University Bundang Hospital (No. BA160-9209-065). Animal care and experiments were performed in compliance with the U.S. National Institutes of Health Guide for the Care and Use of Laboratory Animals. Rats were kept at room temperature with equal lighting control (12 h light/12 h dark). All surgical procedures were conducted at 10 am. 

### 2.2. Experimental Group Assignment

A total of 48 male Sprague-Dawley rats (270–330 g) were randomly assigned to one of 4 groups: (1) sham group (*n* = 12): underwent sham surgery, in which baseline blood pressure was maintained; (2) control group (*n* = 12): mean arterial pressure (MAP) was maintained within 90% of baseline value for 20 min before aortic clamping; 3) mild hypotension group (*n* = 12): MAP was maintained at < 80% of the baseline value for 20 min before aortic clamping; 4) profound hypotension group (*n* = 12): MAP was maintained at < 60% of the baseline value for 20 min before aortic clamping.

### 2.3. Anesthesia and Surgical Preparation

All rats were anesthetized in an acrylic box prefilled with 5.0 vol% of isoflurane in 100% oxygen. Then, maintenance of anesthesia was performed with a facial mask of inhaled 0.5–2.5 vol% isoflurane and oxygen flow of 2 L/min. We placed a polyethylene catheter (PE-50) in the tail artery to monitor distal arterial pressure and inject heparin. A 20-gauge intravenous catheter (BD Insyte®, Becton Dickinson, Sandy, UT, USA) was placed in the right carotid artery to control the proximal arterial pressure. The left femoral artery was cannulated with a 2-Fr. Fogarty catheter (Fogarty Arterial Embolectomy Catheter®, Edwards Lifesciences, Irvine, CA, USA) and 20-gauge catheter (BD Insyte, Becton Dickinson, Sandy, UT, USA), which was connected to a saline-filled external blood reservoir for draining of blood during aortic clamping.

### 2.4. Experimental Protocol

After anesthesia and surgical preparation, hypotension was achieved to each group’s target MAP for 20 min. In the sham and control groups, MAP was maintained for 20 min as that of baseline value without nicardipine administration. In the mild and profound hypotension groups, intravenous nicardipine was administered to achieve the target MAP. Intravenous phenylephrine was used for excessive hypotension (defined as MAP < 90% of each group’s target pressure).

Spinal cord ischemia was achieved using the advancement of the Fogarty catheter. After heparin (150 U) administration, a balloon tipped 2-Fr. Fogarty catheter was advanced into the descending thoracic aorta by the left femoral artery. The catheter tip was placed at the left subclavian artery. The balloon, located at the tip, was inflated with saline (0.05 mL), and simultaneous drainage of blood from the carotid artery into the external reservoir prevented proximal hypertension during the aortic clamping period. An abrupt and sustained decrease in distal arterial pressure (the tail artery pressure) ensured sufficient aortic clamping. After 9 min and 30 sec of aortic clamping, the balloon was deflated, and the blood in reservoir was administered through the catheter placed in the left carotid artery. After that, we removed all catheters and sutured the surgical incision. The rats were placed in their cage after recovering from anesthesia and observed for two days. 

### 2.5. Mean Arterial Pressure Monitoring

MAP was continuously monitored, and normothermia was maintained with a heated blanket. Baseline MAP was measured right after the surgical preparation. MAP during the deliberated hypotensive period was calculated with the average of MAP value recorded every 2 min during the 20 min of deliberated hypotension period. MAP value was also measured at 10 min after the start of reperfusion when the MAP was stable. 

### 2.6. Evaluation of Neurobehavioral Outcomes

The blinded observer evaluated neurobehavioral outcome with hind limb motor function using the motor deficit index (MDI) at 8, 24, and 48 h after reperfusion. The MDI was defined as the sum of the ambulation, placing, and stepping reflex scores. Ambulation was scored as follows: 0, normal (symmetrical and coordinated ambulation); 1, toes flat under body when walking, but ataxia present; 2, knuckle-walking; 3, movement in lower extremities but unable to knuckle-walk; and 4, no movement, drags lower extremities. The placing and stepping reflex was assessed by dragging the dorsum of the hind paw over the edge of a surface, which normally evokes a coordinating lifting and placing (stepping) response. The placing and stepping reflex was measured as follows: 0, normal; 1, weak; and 2, no stepping.

### 2.7. Histopathology

After neurobehavioral outcome evaluation, rats were euthanized with mask-delivered isoflurane. Ice-cold saline was perfused, and the spinal cord of 6 rats in each experimental group was separated and fixed in 10% buffered formalin for 24 h for histopathological examination. The cord segments at the L3-L5 level were embedded in paraffin, and transverse sections were stained with hematoxylin and eosin.

The anterior spinal cord of the paraplegic animals was significantly destroyed with a decrease in normal motor neuron; a blinded observer counted the number of normal motor neurons in each side (right and left) of the anterior horn of the spinal cord (anterior to a line drawn through the central canal perpendicular to the vertebral axis) at × 200 magnification.

### 2.8. Evaluation of the Oxidative Stress and Inflammation

Sections of the lumbar segments (L3, L4, and L5) of the spinal cord were halved along the median sulcus. Each half of the segment was cut into small pieces and homogenized in phosphate-buffered saline using a tube pestle. The homogenate was centrifuged with the addition of acetone at 1500× *g* for 10 min at 4 °C. Oxidative stress was evaluated by measuring the tissue level of malondialdehyde (MDA), the end-product of lipid peroxidation. Tissue MDA level was measured according to the manufacturer’s protocol (MDA-586, OxisResearch, Percipio Biosciences, Burlingame, CA, USA) and was expressed in terms of nmol MDA/g wet tissue. Inflammation level was assessed with tissue tumor necrosis factor-α (TNF-α) level (pg/g prot), which was measured according to the manufacturer’s protocol (Rat TNF-α ELISA Kit, Invitrogen, Thermo Fisher Scientific, Waltham, MA, USA). 

### 2.9. Statistical Analysis

The SPSS 22.0 for Windows (IBM Inc., Armonk, New York, NY, USA) was used for statistical analyses. Data are presented as means ± SD or medians (interquartile range). Data normality was tested with the Shapiro-Wilk test. For MAP, between-group comparisons at each time point were made using analysis of variance (ANOVA), followed by Bonferroni post-hoc comparisons. The outcomes were compared by Kruskal-Wallis test, followed by the Mann-Whitney *U* test. A *p*-value < 0.05 indicated statistical significance. 

## 3. Results

### 3.1. Mean Arterial Pressure 

**Figure 1:**
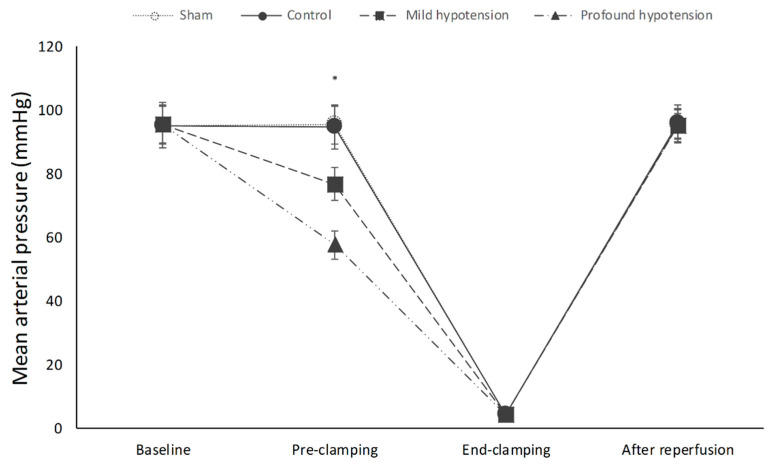
Changes in MAP (mean arterial pressure) during the experiment. There were significant differences in MAP among the 4 groups over time (*p* < 0.001). Bonferroni post-hoc comparisons showed that MAP before aortic clamping was significantly lower in the mild (76.5 ± 5.2 mmHg) and profound hypotension group (57.4 ± 4.3 mmHg) than in the control group (94.5 ± 7.0 mmHg) (*p* < 0.001). MAP before aortic clamping was also lower in the profound hypotension group compared to the mild hypotension group (*p* < 0.001). Results are expressed as mean ± SD.

The change in MAP during the experiment is shown in Figure 1. The baseline MAP values were not different among the three experimental groups. Repeated-measures ANOVA revealed that there were significant differences in MAP among the 4 groups over time (*p* < 0.001). During deliberated hypotension before the aortic clamping period, MAP was significantly lower in the mild and profound hypotension group than in the control group; differences between the two groups (mild and profound hypotension) were also statistically significant (*p* < 0.001). After reperfusion, no difference was found between the four groups.

### 3.2. Neurobehavioral Outcomes

**Figure 2:**
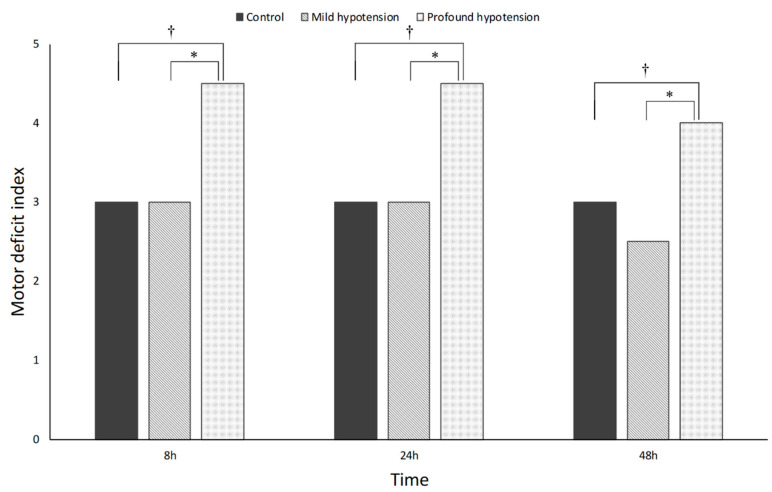
Motor deficit index at 8, 24, and 48 h after reperfusion. The profound hypotension group had significantly higher MDI (motor deficit index) scores than the mild hypotension and control groups. *p* < 0.001 (*,†) by Mann-Whitney *U* test.

Neurobehavioral outcomes, assessed by MDI with hind limb motor function, are presented in Figure 2. No rats in the sham group showed neurobehavioral abnormality. The profound hypotension group had significantly higher MDI scores than the mild hypotension and control groups at 8, 24, and 48 h after reperfusion. However, there were no significant differences in MDI scores between the mild hypotension and control groups 48 h after reperfusion. 

### 3.3. Histopathology

The number of normal motor neurons in the anterior cord is shown in Table 1. The number of normal motor neurons was significantly different among the four groups (*p* < 0.001). No difference between the control and mild hypotension groups was observed (*p* = 0.583). However, the profound hypotension group showed significantly lower number of normal motor neurons compared with the control and mild hypotension groups (*p* < 0.001).

### 3.4. Oxidative Stress and Inflammatory Reaction

**Figure 3:**
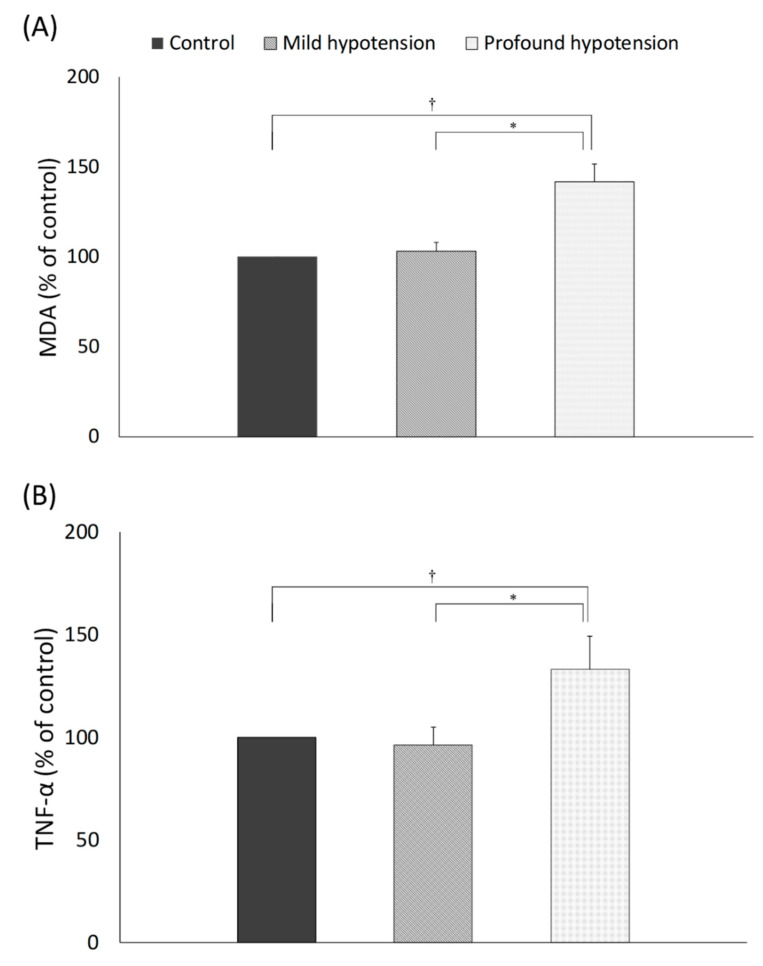
The level of spinal cord tissue MDA (malondialdehyde) (**A**) and TNF-α (tumor necrosis factor-α) (**B**). The levels of MDA and TNF-α were different among the groups (*p* < 0.001). The profound hypotension group showed significantly increased level of MDA and TNF-α compared to other groups. *p* < 0.001 (*,†) by unpaired t-test.

The levels of tissue MDA, an indicator of oxidative stress, were significantly increased in the control, mild hypotension, and profound hypotension groups compared to the sham group (*p* < 0.001) (Figure 3a). The level of tissue MDA was significantly increased in the profound hypotension group compared to the control and mild groups (*p* < 0.001). However, mild hypotension had no influence on the level of tissue MDA compared with the control group (*p* = 0.168). 

The level of TNF-α showed a similar pattern with MDA (Figure 3b). Aortic clamping significantly caused spinal cord inflammation. The level of TNF-α was significantly higher in the control group, mild hypotension, and profound hypotension groups than the sham group (*p* < 0.001). Among the experimental groups, the level of TNF-α was significantly increased in the profound hypotension group compared with the control and mild hypotension groups (*p* < 0.001). However, there was no difference in the level of TNF-α between the control and mild hypotension groups (*p* = 0.337). 

## 4. Discussion

The present study shows that profound hypotension (<60% of baseline blood pressure) can result in deterioration of postoperative neurological function. To the best of our knowledge, this is the first study that identifies the effect of degrees of deliberated hypotension during the pre-aortic clamping period on spinal cord ischemic injury in acute aortic syndrome. 

A wide variety of pharmacologic agents is used to induce hypotension in acute aortic syndrome [9]. In the present study, nicardipine was selected as a hypotensive agent as it does not cause tachycardia, which is harmful to aortic wall pressure. Furthermore, nicardipine is commonly used in acute aortic syndrome patients before surgical repair [14,15].

Previous data on the effects of controlled hypotension on spinal cord ischemia in acute aortic syndrome are scarce; however, several reports were in concordance with our finding. Our results are in line with a previous report from patients who underwent TEVAR. The European Registry of Endovascular Aortic Repair Complications has reported a direct correlation between a prolonged period of intraoperative hypotension (>5 min) and symptomatic spinal cord ischemia [4]. Although the exact degree or duration of hypotension was not documented in that study, the results implicate the importance of intraoperative hypotension in spinal cord ischemic injury.

One case report presented the concomitant fall in mean arterial pressure and spinal cord near-infrared spectroscopy (NIRS) value [16]. Spinal NIRS is reported to be able to detect against regional spinal cord ischemia [17]. Although there is no laboratory data regarding the effect of deliberate hypotension on spinal cord ischemia, laboratory data suggesting the possible deleterious effect of controlled hypotension on cerebral ischemia exist. In a monkey model of middle cerebral artery occlusion, deliberate hypotension to a mean blood pressure of 45–50 mmHg resulted in significant aggravation of ischemic injury [12]. Similarly, in a rat model of middle cerebral artery occlusion, deliberate hypotension significantly increased cerebral infarct zone size, irrespective of the hypotensive regimen [13]. Blood pressure reduction before recanalization is associated with aggravated ischemic injury in patients with cerebral infarct [18,19].

Unsaturated fatty acids in the biological membrane of the neuron are vulnerable to free radical and lipid peroxidation, which mediates oxidative stress [20]. Oxidative stress plays an important role in secondary tissue injury following reperfusion [21,22]. MDA, the end product of lipid peroxidation, is directly related with the level of free radicals. This study demonstrated that profound hypotension led to increased levels of MDA compared to other groups, suggesting extensive reperfusion injury. Several animal studies using a rat model of spinal cord injury also demonstrated that the level of MDA increased after spinal cord injury, regardless of type of injury, including trauma, radiation, or ischemia-reperfusion [23,24,25].

Inflammation is another main cause of secondary damage after spinal cord injury [26]. Reperfusion paradoxically exacerbates tissue damage since reperfusion may not restore blood supply to ischemic tissue immediately, an effect known as ‘no-reflow phenomenon’. This phenomenon may activate autoimmune, innate immune, and adaptive immune systems, which induce cell death via necrosis and apoptosis [27]. Cell necrosis and apoptosis contribute to the production of inflammatory factors, such as IL-1β, IL-10, and TNF-α, which enhance phagocytosis, leading to more extensive reperfusion injury [26]. Our experiments confirmed the previous results [28,29] in that the profound hypotension group showed higher levels of TNF-α than other groups. Thus, it can be reasonably assumed that ischemia-reperfusion induced by profound hypotension leads to extensive oxidative stress and inflammation in the spinal cord.

There are several limitations in this study. First, to clarify the effect of deliberate hypotension, we did not use any other adjuvant protective strategy, such as hypothermia or cerebrospinal fluid drainage [30,31]. Thus, when other adjuvant therapy is adopted, the safety margin of the deliberate hypotension might be different from the present study’s results. Second, MAP was targeted and controlled to induce hypotension in this study. It was reported that spinal cord perfusion pressure depends on MAP [32]. However, other hemodynamic parameters, such as systolic blood pressure, diastolic blood pressure, or stroke volume, may provide useful information about the relationship between hemodynamics and spinal cord ischemia.

## 5. Conclusions

In conclusion, the present study using an animal model suggests that excessive degrees of deliberate hypotension (<60% MAP) can aggravate spinal cord ischemia following aorta surgery. Meticulous selection of degree and duration of hypotension is necessary to prevent aggravation of spinal cord ischemia in patients with acute aortic syndrome.

## Figures and Tables

**Table 1:** The number of normal motor neuron in the anterior spinal cord.

Sham Group	Control Group	Mild Hypotension Group	Profound Hypotension Group	*p* Value
16.9 (1.3) ^†^	11.9 (1.7)	11.3 (1.7)	7.5 (1.7) ^‡^	<0.001

Data are expressed as mean (standard deviation). ^†^ *p* < 0.001 vs. control, mild hypotension, and profound hypotension group, ^‡^ *p* < 0.001 vs. sham, control, and mild hypotension group.
